# Negative Affect and Excessive Alcohol Intake Incubate during Protracted Withdrawal from Binge-Drinking in Adolescent, But Not Adult, Mice

**DOI:** 10.3389/fpsyg.2017.01128

**Published:** 2017-07-06

**Authors:** Kaziya M. Lee, Michal A. Coehlo, Noah R. Solton, Karen K. Szumlinski

**Affiliations:** ^1^Department of Psychological and Brain Sciences, University of California, Santa BarbaraSanta Barbara, CA, United States; ^2^Department of Molecular, Cellular and Developmental Biology and The Neuroscience Research Institute, University of California, Santa Barbara, Santa BarbaraCA, United States

**Keywords:** binge drinking, adolescence, Group 1 metabotropic glutamate receptors, receptors, anxiety, depression, alcoholism

## Abstract

Binge-drinking is common in underage alcohol users, yet we know little regarding the biopsychological impact of binge-drinking during early periods of development. Prior work indicated that adolescent male C57BL6/J mice with a 2-week history of binge-drinking (PND28-41) are resilient to the anxiogenic effects of early alcohol withdrawal. Herein, we employed a comparable Drinking-in-the-Dark model to determine how a prior history of binge-drinking during adolescence (EtOH^adolescents^) influences emotionality (assayed with the light-dark box, marble burying test, and the forced swim test) and the propensity to consume alcohol in later life, compared to animals without prior drinking experience. For additional comparison, adult mice (EtOH^adults^) with comparable drinking history (PND56-69) were subdivided into groups tested for anxiety/drinking either on PND70 (24 h withdrawal) or PND98 (28 days withdrawal). Tissue from the nucleus accumbens shell (AcbSh) and central nucleus of the amygdala (CeA) was examined by immunoblotting for changes in the expression of glutamate-related proteins. EtOH^adults^ exhibited some signs of hyperanxiety during early withdrawal (PND70), but not during protracted withdrawal (PND98). In contrast, EtOH^adolescents^ exhibited robust signs of anxiety-l and depressive-like behaviors when tested as adults on PND70. While all alcohol-experienced animals subsequently consumed more alcohol than mice drinking for the first time, alcohol intake was greatest in EtOH^adolescents^. Independent of drinking age, the manifestation of withdrawal-induced hyperanxiety was accompanied by reduced Homer2b expression within the CeA and increased Group1 mGlu receptor expression within the AcbSh. The present data provide novel evidence that binge-drinking during adolescence produces a state characterized by profound negative affect and excessive alcohol consumption that incubates with the passage of time in withdrawal. These data extend our prior studies on the effects of subchronic binge-drinking during adulthood by demonstrating that the increase in alcoholism-related behaviors and glutamate-related proteins observed in early withdrawal dissipate with the passage of time. Our results to date highlight a critical interaction between the age of binge-drinking onset and the duration of alcohol withdrawal in glutamate-related neuroplasticity within the extended amygdala of relevance to the etiology of psychopathology, including pathological drinking, in later life.

## Introduction

Adolescence is a critical period of accelerated neurodevelopment, which occurs between the ages of approximately 11–21 years in humans and conservative estimates of adolescence in rodents range from postnatal days (PNDs) 28–42 (Spear, 2000, 2010; Laviola et al., 2003). During adolescence, there is a dramatic reduction of gray matter as the cortex undergoes synaptic pruning, and a proliferation of white matter from ongoing myelination of axons, leading to extensive remodeling of the structure and function of the brain (e.g., Sowell et al., 2003; Gogtay et al., 2004). These processes are essential for refining excitatory and inhibitory connectivity and stabilizing synapses within corticofugal projections that exert control over subcortical hyperactivation (Casey et al., 2011; Sturman and Moghaddam, 2011; Arain et al., 2013). Thus, adolescents typically exhibit increased impulsivity, sensation/novelty seeking, risk-taking, and mood swings, compared to adults (Casey et al., 2008; Sturman and Moghaddam, 2011; Spear and Swartzwelder, 2014). Drug experimentation is also common during the adolescent stage of development, with alcohol being the most commonly used substance among adolescents (Kelley et al., 2004; Lopez et al., 2008). Indeed, underage alcohol-drinking is a serious public health concern, with 7.7 million individuals between the ages of 12–20 reporting drinking alcohol within the past month (published by the Center for Behavioral Health Statistics and Quality, 2016). Over 90% of alcohol consumed by underage drinkers is in the form of binge-drinking episodes (National Institute on Alcohol Abuse, and Alcoholism [NIAAA], 2017), i.e., consumption sufficient to achieve a blood alcohol concentration (BACs) ≥80 mg/dL (approximately 4–5 drinks) in a 2-h period (National Institute on Alcohol Abuse, and Alcoholism [NIAAA], 2004). Additionally, research has consistently shown that adolescent drinking is one of the strongest predictors of substance abuse problems and addiction later in life (Grant and Dawson, 1997; Chassin et al., 2002; Tapert and Schweinsburg, 2005).

In both humans and animal models, adolescents typically consume larger quantities of alcohol than adults per drinking episode and adolescents also respond differently to alcohol than their adult counterparts (White et al., 2002; Spear and Varlinskaya, 2005; Novier et al., 2015). Adult drinkers often show pronounced signs of acute withdrawal following a binge episode, including headaches, anxiety, agitation, lethargy, gastrointestinal distress, in severe cases even withdrawal-induced seizures (Knapp et al., 1998). In contrast, both clinical and preclinical data show that adolescents tend to be less sensitive than adults both to the negative properties of acute intoxication such as sedation, motor impairment, and hypothermia, as well as the ‘hangover’ symptoms seen in adults during withdrawal (Little et al., 1996; White et al., 2002; Doremus et al., 2003; Varlinskaya and Spear, 2004; Anderson et al., 2010; Schramm-Sapyta et al., 2010). At the same time, adolescents show increased sensitivity to the pleasurable, reinforcing properties of alcohol such as positive reward and social facilitation (Pautassi et al., 2008; Ristuccia and Spear, 2008; Doremus-Fitzwater et al., 2010). Blunting of the aversive consequences that typically serve as negative feedback to inhibit excessive consumption, along with enhancement of the positive incentive properties of alcohol, are theorized to promote high alcohol consumption in both human and animal adolescents (Spear and Varlinskaya, 2005).

Binge-drinking is the most toxic pattern of excessive alcohol consumption and has been shown to produce a ‘kindling’ effect (Ballenger and Post, 1978; Becker, 1998), whereby repeated cycles of acute intoxication followed by periods of abstinence intensify withdrawal-induced neurotoxicity (Begleiter and Porjesz, 1979; Overstreet et al., 2002; Duka et al., 2004). Frequent binge-drinkers can rapidly develop tolerance to the subjective intoxicating effects of alcohol, leading to an escalation of intake and brain exposure to harmful concentrations of alcohol (Tabakoff et al., 1986; Hoffman and Tabakoff, 1989; Gruber et al., 1996). This is particularly concerning, as research suggests that adolescents are uniquely susceptible to neurotoxic insult resulting from chronic alcohol exposure and can suffer potentially life-long dysfunction resulting from perturbed maturation of prefrontal control over subcortical circuitry, particularly within regions involved in emotionality (Casey and Jones, 2010; Crews et al., 2016).

Studies have revealed persistent alcohol-induced neurobiological changes within the extended amygdala – the subcortical macrostructure integrally involved in governing diverse emotional states (Alheid, 2003; Jennings et al., 2013; Shackman and Fox, 2016). The extended amygdala consists of the central nucleus of the amygdala (CeA), bed nucleus of the stria terminalis (BNST), and shell subregion of the nucleus accumbens (AcbSh). These structures are highly vulnerable to drug-induced plasticity and dysregulation within the extended amygdala circuitry is known to underlie many of the negative reinforcing properties of withdrawal that fuel the cycle of addiction (reviewed in Koob, 2003; Baker et al., 2004). Mood disorders such as anxiety and depression, are also thought to be related to abnormal corticofugal development resulting in insufficient regulatory control over subcortical regions involved in emotion and motivation, for example the AchSh and CeA (Andersen and Teicher, 2008). Common underlying neuropathology could account for the high comorbidity between alcohol abuse and mood disorder, which is especially prominent amongst those with a history of drinking during adolescence. In fact, adolescent alcohol use disorder is one of the strongest predictors of major depressive disorder in adulthood (Grant and Dawson, 1997; Briere et al., 2014).

Consistent with existing human and animal research, previous work from our lab has demonstrated that adolescent mice exhibit minimal signs of negative affect during early (24 h) withdrawal, and are also resistant to changes in protein expression within the Acb (Lee et al., 2016) using the Drinking-in-the-Dark (DID) animal model of voluntary binge-drinking (Rhodes et al., 2005, 2007; Thiele and Navarro, 2014). For example, we demonstrated recently that, in contrast to adult binge-drinking mice that exhibit robust anxiety-like behavior during early (24 h) withdrawal across several conventional behavioral tests of negative affect (e.g., light-dark shuttle box, novel object encounter, Porsolt swim test, elevated plus-maze), adolescent binge-drinking mice resemble water-drinking controls (Lee et al., 2015, 2016). In the present study, we sought to expand these findings to assess the adult consequences of adolescent binge-drinking on negative affect and subsequent alcohol-drinking. Based on the human literature (Grant and Dawson, 1997; Chassin et al., 2002; Briere et al., 2014), we predicted that when tested during adulthood (i.e., in protracted withdrawal), adolescent drinkers would show signs of alcohol-induced negative affect and increased alcohol consumption. Other studies of this nature have typically employed alcohol-naïve animals as the control group; however, we also wanted to compare adolescent drinkers to animals with equivalent drinking experience during adulthood, in order to specifically isolate the unique effects of alcohol during adolescence from the non-age-dependent effects of alcohol, more generally. Based on the high-risk nature of adolescent binge-drinking reported clinically, we speculated that the withdrawal-induced hyper-anxiety manifested in adulthood would be more pronounced in animals with a prior history of binge-drinking during adolescence, than in animals with a prior history of drinking during adulthood. To complement the behavioral data, we also collected brain tissue samples from the AcbSh and CeA for immunoblotting, as these extended amygdala structures exhibit hyperactivity in adult mice during withdrawal from binge-drinking (Lee et al., 2015), as well as increases in protein indices of glutamate transmission that promote binge-alcohol intake (e.g., Cozzoli et al., 2009, 2012, 2014, 2015). We sampled tissue also from the adjacent nucleus accumbens core (AcbC) and the basolateral amygdala (BLA) to examine the subregional specificity of any observed protein effects. These adjacent subregions share connectivity and proximity with the extended amygdala but are not considered parts of this macrosystem, thus enabling us to determine whether or not any observed changes in protein expression were specific to the extended amygdala. If adult and adolescent drinkers do indeed show distinct withdrawal phenotypes, these differences could be reflected in divergent alcohol-induced protein changes within extended amygdala structures.

## Materials and Methods

Experimental procedures were similar to those in our previous studies (Lee et al., 2015, 2016) and are briefly summarized below. All experiments were conducted in compliance with the National Institutes of Health Guide for Care and Use of Laboratory Animals (NIH Publication No. 80–23, revised 2014) and approved by the IACUC of the University of California, Santa Barbara.

### Subjects

**FIGURE 1:**
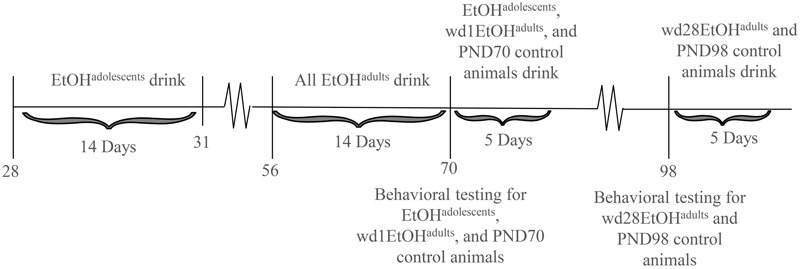
Procedural time-line of the experiments. Summary of the timing of the binge-drinking and testing procedures for comparing the protracted effects of a history of binge-drinking during adolescence (EtOH^adolescents^) or adulthood (EtOH^adults^) upon behavioral measure of negative affect and subsequent alcohol intake. wd1 and wd28 denote, respectively, 1 and 28 days withdrawal.

The animals used in this study were male C57BL/6J mice (Jackson Laboratories, Sacramento, CA, United States). Animals were housed in groups of 4 in standard Plexiglas cages, in a temperature-controlled vivarium (23°C), under a 12 h reverse light/dark cycle (lights off at 10 am). Food and water were available *ad libitum*, except during the 2 h alcohol-drinking period. Adolescent drinkers (EtOH^adolescents^) began drinking at PND28, spanning the approximate period of early-mid adolescence in mice (Spear, 2000; Brust et al., 2015), and underwent behavioral testing in adulthood on PND70, after 28 days withdrawal (i.e., protracted withdrawal). Adult drinkers (EtOH^adults^) were PND56 at drinking onset and consisted of two subgroups: one group was behaviorally tested at PND70, after 1 day withdrawal (wd1EtOH^adults^), to match the age of the aforementioned EtOH^adolescents^ mice and control for known age-related differences in basal behavior and protein expression (Spear, 2010). In a follow-up experiment, an additional group of adult mice was added to the study and tested for behavior on PND98 (i.e., after 28-days withdrawal; wd28EtOH^adults^), to control for the effects of a 28-days withdrawal period upon behavior/protein expression. All control animals (PND70 and PND98) received only water prior to behavioral testing. Sample sizes were *n* = 9 for all groups. The experimental timeline for behaviorally tested animals is summarized in **Figure 1**.

A separate cohort of animals (*n* = 12/group) was used to generate brain tissue for immunoblotting, as a previous study from our laboratory showed that behavioral testing procedures induced cellular activation within Acb subregions (Lee et al., 2015). These animals were subjected to the same drinking procedures as the animals in the behavioral experiment, but were sacrificed on PND70 or PND98 to obtain brain tissue, in lieu of behavioral testing.

### Drinking-in-the-Dark (DID) Procedures

#### Initial Alcohol Exposure

All alcohol-experienced animals were exposed to 14 consecutive days of binge-drinking under our 4-bottle DID procedures (Lee et al., 2016). Alcohol-access was restricted to 14 days in order to correspond with the estimated length of early-mid adolescence in mice (Spear, 2000), when developmental changes are most prolific (Spear, 2010). The DID protocol is a widely accepted model of binge-drinking that has been shown to elicit high voluntary alcohol consumption in laboratory animals (Rhodes et al., 2005; Crabbe et al., 2009). Each day prior to the drinking period, animals were separated into individual cages and allowed to acclimate for approximately 45 min. Beginning 3 h into the dark phase of the circadian cycle, the peak time of daily fluid intake (Rhodes et al., 2005), animals were given simultaneous access to 5, 10, 20, and 40% (v/v) unsweetened ethanol solutions for 2 h. The positioning of the bottles on the cage was randomized each day. Expanding the traditional 1-bottle DID protocol to include 4 bottles of differing concentration has been shown to elicit even higher voluntary intakes (Henniger et al., 2002; Tordoff and Bachmanov, 2003; Gustafsson and Nylander, 2006; Cozzoli et al., 2014), as animals are able to sample from all the bottles and consume whichever concentration they find most palatable. This being said, the immunoblotting results for the CeA that ensued from our study of mice drinking under the 4-bottle procedure (see below) prompted us to conduct a follow-up immunoblotting study in which mice were presented with a single bottle containing 20% (v/v) alcohol for 2 h/day for 14 days. In either case, the amount of alcohol consumed each day was calculated by bottle weight immediately before and after the drinking period.

#### Blood Alcohol Sampling

Submandibular blood samples were collected on drinking day 10, immediately following the 2-h drinking period. The scheduling of the blood sampling was selected to ensure that the animals’ intakes had stabilized, while also allowing ample time for recovery prior to behavioral testing. BACs were determined using an Analox alcohol analyzer (model AM1, Analox Instruments United States, Lunenburg, MA, United States).

#### Subsequent Drinking in Adulthood

Beginning approximately 24 h following behavioral testing, all animals, including previously alcohol-naïve water drinkers, were subjected to 5 additional days of DID procedures in order to relate prior alcohol experience, age of first exposure, and affective state to alcohol consumption in adulthood.

### Behavioral Testing

Behavioral testing consisted of the marble burying test, which was followed by the Porsolt forced swim test (FST). Both of these procedures were demonstrated to be particularly sensitive to the effects of alcohol withdrawal in our previous studies of mice (Lee et al., 2015, 2016). The order of testing was based on recommendations from our IACUC discouraging additional testing following the FST to allow animals to fully recover.

#### Marble Burying

The marble-burying test was used as a measure of anxiety-induced defensive burying, as an increase in burying-related behavior serves as an index of anxiety (Young et al., 2006; Umathe et al., 2008) In our paradigm, 12 square glass pieces (2.5 cm^2^×1.25 cm tall) were placed in the animals’ home cage, six at each end. Animals were then left undisturbed for 15 min and video recorded for later analysis. At the end of the trial, a blind observer recorded the number of marbles at least 75% buried. Later, a blind observer reviewed the video footage and the latency to begin burying and the total time spent burying was recorded using a stopwatch.

#### Porsolt Forced Swim Test

The FST is a common measure of depression-like behaviors in laboratory animals, based on changes in active swimming (Porsolt et al., 1977a,b, 2001). Each animal was placed into an 11-cm diameter cylindrical container filled with room-temperature water such that animals were unable to touch the bottom. The latency to first exhibit immobility (defined as no horizontal or vertical displacement of the animal’s center of gravity for 5^++^s), total time spent immobile, and the numbers of immobile episodes were monitored during a 6-min period using AnyMaze^TM^ tracking software (Stoelting Co., Wood Dale, IL, United States).

#### Sucrose Preference Test

The sucrose preference test is a common assay of anhedonia (e.g., Serchov et al., 2016), used to model depression in laboratory animals (Katz, 1982; Willner et al., 1992). Upon conclusion of the marble burying and FST, animals were returned to the colony room and presented with overnight access to 2 identical sipper tubes, one containing 5% sucrose and the other containing plain water. Bottles were weighed prior to being placed on the home cage at approximately 16:00 h and again after removal at 09:00 h the following day. Change in bottle weight was used to determine the volume consumed and a relative sucrose preference was calculated as the volume of sucrose consumed/total fluid volume consumed.

### Brain Tissue Collection

Animals in the immunoblotting study were rapidly decapitated approximately 24 h following the final alcohol presentation to mirror the time-frame of the behavioral testing. Brains were removed and cooled on ice, then sectioned in 1 mm-thick coronal slice at the level of the striatum and amygdala. The AcbSh and CeA were bilaterally sampled from the slice located approximately 1.18 mm and -1.22 mm relative to Bregma, respectively, as shown in the mouse brain atlas of Paxinos and Franklin (2004), using a 18-gauge biopsy needle (depicted in **Figures 6, 7**).

### Immunoblotting

Western blotting was performed on whole tissue homogenates from the AcbSh and AcbC (AP +1.18 mm), and CeA and BLA (AP -1.34 mm) (location relative to bregma, as depicted in Paxinos and Franklin, 2004) following procedures identical to those described in Lee et al. (2016). The following primary antibodies and concentrations were used: mGlu1 (Synaptic Systems, Göttingen, Germany; 1:1000 dilution), mGlu5 (Millipore, Temecula, CA, United States; 1:1000 dilution), Homer2b (Millipore, Temecula, CA, United States; 1:1000 dilution), and calnexin (Enzo Life Sciences, Farmingdale, NY, United States; 1:1000 dilution) for standardization. Homer2b is a postsynaptic density scaffolding protein that regulates signaling of Group 1 metabotropic glutamate receptors (mGluRs) (Szumlinski et al., 2005). Together, these proteins were selected for study based on our laboratory’s prior work identifying them as relevant to alcohol-induced neuroplasticity (Szumlinski et al., 2005, 2007, 2008; Cozzoli et al., 2009, 2012, 2014, 2015; Obara et al., 2009; Goulding et al., 2011; Lum et al., 2014; Lee et al., 2015; Quadir et al., 2016).

### Statistical Analysis

Alcohol intake data from the 14-day drinking period were analyzed with a repeated measures analysis of variance (ANOVA), with drinking age (EtOH^adolescents^ or wd1EtOH^adults^) as the between-subjects factor and day (14 days) as the within-subjects repeated measure to screen for potential group differences in alcohol consumption, which could confound alcohol-induced behavioral and neurobiological changes. The 5-day intake data was similarly analyzed with a drinking age (EtOH^adolescents^, wd1EtOH^adults^, or no prior experience) X day (5) repeated-measures ANOVA. A repeated measures ANOVA was also used determine if there was an effect of age/prior alcohol experience on the preference for a particular alcohol concentration, with drinking age (EtOH^adolescents^, wd1EtOH^adults^, or no prior experience) as the between-subjects factor and both day (14 or 5 levels) and concentration (5, 10, 20, or 40%) as the within-subjects factors.

Behavioral data for animals tested at PND70 were analyzed using between-subjects ANOVAs, with drinking (EtOH^adolescents^ or wd1EtOH^adults^) as the between-subjects factor, and Tukey’s *post hoc* comparisons when appropriate; α = 0.05. All comparisons between wd28EtOH^adults^ and age-matched control animals were conducted using independent samples *t*-tests with Bonferroni corrections for multiple comparisons, as these animals were run as a separate follow-up to the animals tested at PND70. Paired-samples *t*-tests were used to compare the average consumption during the first and second rounds of drinking in alcohol-experienced animals.

The immunoblotting data for the animals subjected to our 4-bottle-choice drinking procedures were analyzed using a drinking age (EtOH^adolescents^, wd1EtOH^adults^, or no prior experience) univariate ANOVA, while that for the animals subjected to our single-bottle procedure were analyzed using unpaired-samples *t*-tests. For all analyses, statistical outliers were identified using the ±1.5^∗^IQR rule and omitted from analyses. There were no statistical outliers excluded from the behavioral data. Outlier exclusion resulted in n’s of 10–12 per group for the immunoblotting data (the specific n’s for individual analyses are reported in the figure legends). All statistics and calculations were performed using SPSS v.21 statistical software.

## Results

### 14-Day Alcohol Consumption

Although the repeated measures ANOVA showed no between-subjects differences in the total amount of alcohol consumed by EtOH^adolescents^, wd1EtOH^adults^, and wd28EtOH^adults^ across the initial 14-day drinking period [*F*(2,24) = 0.14, *p* = 0.87], there was a significant age × day within-subjects interaction [*F*(78,845) = 6.11, *p* < 0.001]. Further analysis revealed that over days 1–7, wd1EtOH^adults^ drank more alcohol than EtOH^adolescents^ (*p* = 0.001) but the converse occurred over days 8–14 (*p* < 0.001). This shift was reflected by a similar drinking-age × concentration × day interaction [*F*(39,624) = 4.62, *p* < 0.001] for concentration preference, with EtOH^adolescents^ exhibiting greater preference for lower concentration during the first week and a shift to a preference for higher concentration during the second week compared to wd1EtOH^adults^ (**Table 1**). The repeated measures ANOVA also showed a significant drinking-age × concentration interaction [*F*(3,48) = 3.317, *p* = 0.028] and *post hoc* analysis revealed that wd1EtOH^adults^ had a lower preference for the 5% concentration and a higher preference for the 20% concentration compared to EtOH^adolescents^ (*p* = 0.03 and *p* = 0.049, respectively). There was a trend toward higher preference for the 40% in adolescents compared to adults (*p* = 0.078). During the subsequent 5-day drinking period, there were no significant main effects or interactions between age/prior alcohol experience or concentration (*p*’s > 0.10).

**Table 1:** Summary of group differences in the preference for different alcohol concentrations during the 2-week drinking period.

Day 1	EtOH^adolescents^ had a higher 5% and lower 40% preference, compared to wd1EtOH^adults^ (*p* < 0.001 and *p* = 0.014, respectively)
Day 2	EtOH^adolescents^ had a higher 10% and a lower 20% preference, compared to wd1EtOH^adults^ (*p* = 0.047 and *p* = 0.003, respectively)
Day 3	EtOH^adolescents^ had a higher 40% preference, compared to wd1EtOH^adults^ (*p* = 0.037)
Day 4	No differences
Day 5	EtOH^adolescents^ had a higher 5 and 10% (*p* = 0.009 and *p* = 0.007, respectively), but lower 20 and 40% preference, compared to wd1EtOH^adults^ (*p* = 0.005 and *p* < 0.001, respectively)
Day 6	No differences
Day 7	EtOH^adolescents^ had a higher 5%, but lower 20%, preference, compared to wd1EtOH^adults^ (*p* = 0.002 and *p* = 0.014, respectively)
Day 8	No differences
Day 9	EtOH^adolescents^ had a higher 5%, but lower 10%, preference, compared to wd1EtOH^adults^ (*p* = 0.005 and *p* = 0.022, respectively)
Day 10	EtOH^adolescents^ had a higher 5% preference, compared to wd1EtOH^adults^ (*p* = 0.001)
Day 11	EtOH^adolescents^ had a lower 10%, but higher 40%, preference, compared to wd1EtOH^adults^ (*p* = 0.002 and *p* = 0.037, respectively).
Day 12	EtOH^adolescents^ had a higher 40% preference, compared to wd1EtOH^adults^ (*p* < 0.001)
Day 13	EtOH^adolescents^ had a higher 10%, lower 20%, higher 40% (*p* = 0.048, *p* = 0.002, and *p* = 0.014), compared to wd1EtOH^adults^
Day 14	EtOH^adolescents^ had a lower 10 and 20%, but higher 40%, preference, compared to wd1EtOH^adults^ (*p* = 0.04, *p* = 0.031, *p* < 0.001)

*Results of *post hoc* analysis of the day × age × concentration interaction [*F*(39,624) = 4.62, *p* < 0.001] in EtOH^*adolescents*^ and wd1EtOH^*adults*^; *n* = 9/group.*

There were no differences in alcohol intake amongst the animals used for tissue collection [*F*(2,32) = 0.39, *p* = 0.68] and an overall analysis of all alcohol-drinking animals revealed no differences between cohorts used for behavioral testing or tissue collection [*F*(5,56) = 0.69, *p* = 0.63; summarized in **Table 2**].

**Table 2:** Summary of the average total alcohol intake exhibited by mice with a 14-day history of binge-drinking during adolescence (EtOH^adolescents^), or during adulthood (wd1 or wd28EtOH^adults^).

	Behavioral testing animals	Immunoblotting animals
EtOH^adolescents^	4.16 ± 0.10	4.48 ± 0.15
wd1EtOH^adults^	4.05 ± 0.14	4.46 ± 0.08
wd28EtOH^adults^	4.12 ± 0.10	4.40 ± 0.12

*Note that there were no significant group differences in alcohol intake across the 14-day drinking period.*

### Blood Alcohol Concentrations

**FIGURE 2:**
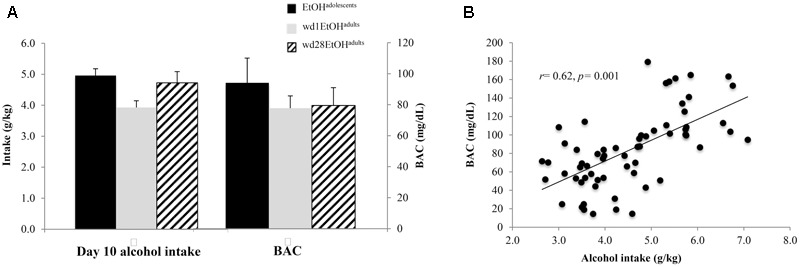
Day 10 BAC sampling. **(A)** Average alcohol intake and BAC by group, averaged across both behavioral testing and immunoblotting animals (*n* = 21/group). **(B)** Alcohol intake was significant correlated with BAC. Data depict all alcohol-drinking animals (*n* = 63).

As the ANOVA revealed no significant differences in alcohol consumption between behavioral testing and immunoblotting animals, day 10 intakes and BACs were collapsed across both cohorts within each drinking group (**Figure 2A**). On day 10 of drinking, EtOH^adolescents^ consumed an average 4.96 ± 0.21 g/kg of alcohol with a resulting BAC of 94.18 ± 9.25 mg/dL; wd1EtOH^adults^ consumed an average of 3.93 ± 0.22 g/kg with a resulting BAC of 77.73 ± 8.46, and wd28EtOH^adults^ consumed an average of 4.72 ± 0.25 g/kg with a resulting BAC of 79.47 ± 9.05 mg/dL. BAC was significantly correlated with alcohol consumption when sampled on day 10 of drinking (*r* = 0.62, *p* = 0.001, *n* = 63; **Figure 2B**).

### Sucrose Preference

**FIGURE 3:**
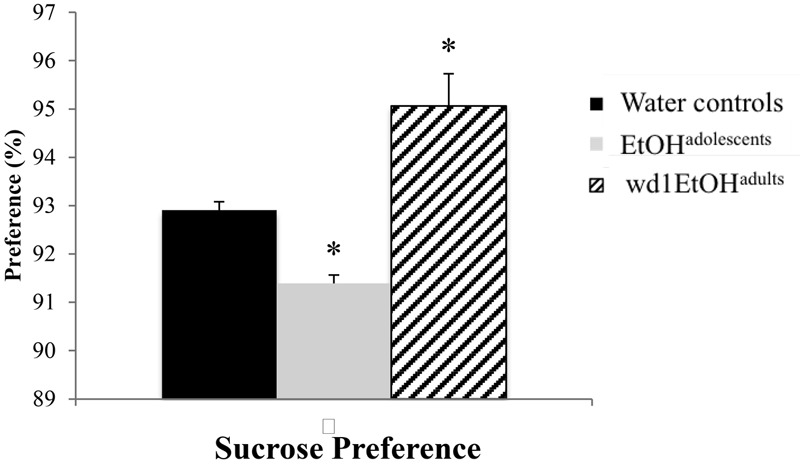
Altered sucrose preference following alcohol drinking. EtOH^adolescents^ showed significantly reduced sucrose preference compared to water control animals while wd1EtOH^adults^ showed increased preference. ^∗^*p* < 0.05 vs. water controls. Data represent mean + SEM, *n* = 9/group.

The ANOVA showed significant group differences in sucrose preference [*F*(2,24) = 20.01, *p* < 0.001; **Figure 3**] and *post hoc* analysis revealed that while wd1EtOH^adults^ showed increased sucrose preference (*p* = 0.003 compared to alcohol-naïve control animals), EtOH^adolescents^ showed decreased preference (*p* = 0.04).

### Marble Burying

**FIGURE 4:**
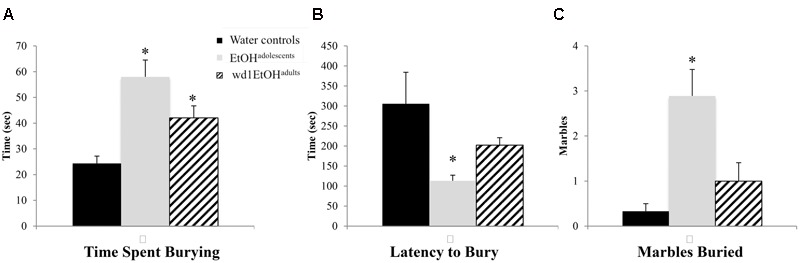
Increased marble burying following alcohol drinking. **(A)** Both EtOH^adolescents^ and wd1EtOH^adilts^ spent significantly more time burying marbles compared to control animals. **(B)** EtOH^adolescent^ also had a shorter latency to start burying and **(C)** buried more marbles overall compared to both control animals and wd1EtOH^adults^. ^∗^*p* < 0.05 vs. water controls. Data represent mean + SEM, *n* = 9/group.

In the marble burying test, there were significant group differences in total time spent burying [*F*(2,24) = 11.82, *p* < 0.001; **Figure 4A**], the latency to start burying [*F*(2,24) = 4.15, *p* = 0.028; **Figure 4B**], and total number of marbles buried [*F*(2,24) = 9.76, *p* = 0.001; **Figure 4C**]. Both wd1EtOH^adults^ and EtOH^adolescents^ spent more time burying compared to water controls (*p* = 0.04 and *p* < 0.001, respectively). EtOH^adolescents^ also had a shorter latency to start burying (*p* = 0.022) and buried more marbles overall (*p* = 0.001). However, wd1EtOH^adults^ did not differ significantly from controls on these factors (*p*’s > 0.1). There were no differences between wd28EtOH^adults^ and age-matched control animals on any behavioral factor tested (*p*’s > 0.10, non-significant results are summarized in **Table 3**).

**Table 3:** Behavioral results from adult drinkers during protracted withdrawal.

	PND98 water controls	wd28EtOH^adults^
Marbles buried	1.44 ± 0.60	2.00 ± 0.40
Time spent burying (s)	32.67 ± 4.56	40.51 ± 5.32
Latency to bury (s)	119.44 ± 16.19	105.78 ± 14.26
FST immobile episodes	20.77 ± 1.19	19.77 ± 0.92
Time spent immobile (s)	111.86 ± 6.13	110.35 ± 11.34
Latency to first immobility (s)	51.53 ± 7.09	54.68 ± 3.70
Sucrose preference	93.87 ± 0.21	93.44 ± 0.25
5-day drinking average (g/kg)	3.09 ± 0.17	3.63 ± 0.25

*When tested 3 weeks following the end of their 14-day drinking session, no significant differences in behavior was observed between mice with a history of binge alcohol-drinking during adulthood (wd28EtOH^*adults*^) and their age-matched water controls. Data represent mean ± SEM, *n* = 9/group.*

### Forced Swim Test

**FIGURE 5:**
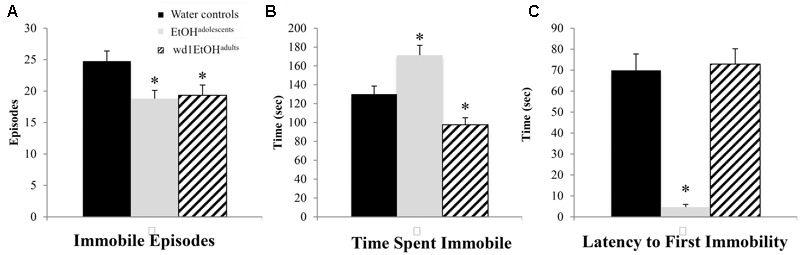
Altered FST behavior following alcohol drinking. **(A)** Both EtOH^adolescents^ and wd1EtOH^adilts^ had fewer immobile episodes than water controls. **(B)** EtOH^adolescents^ spent more time immobile compared to control animals, while wd1EtOH^adults^ spent less time immobile. **(C)** EtOH^adolescents^ had a shorter latency to first immobility, although wd1EtOH^adults^ did not differ significantly from control animals. ^∗^*p* < 0.05 vs. water controls. Data represent mean + SEM, *n* = 9/group.

In the FST, there were group differences found for the number of immobile episodes [*F*(2,24) = 3.94, *p* = 0.033; **Figure 5A**], total time spent immobile [*F*(2,24) = 17.49, *p* < 0.001; **Figure 5B**], and the latency to first immobility [*F*(2,24) = 38.81, *p* < 0.001; **Figure 5C**]. Both wd1EtOH^adults^ and EtOH^adolescents^ had significantly fewer immobile episodes (*p* = 0.04 and *p* = 0.02, respectively) compared to control animals, but EtOH^adolescents^ spent significantly more time immobile (*p* = 0.008), while adults spent less (*p* = 0.04). EtOH^adolescents^ also had a shorter latency to first immobility (*p* < 0.001) but wd1EtOH^adults^ did not (*p* > 0.20). Despite having fewer immobile episodes, EtOH^adolescents^ spent more time immobile, compared to control animals, thus reflecting an overall increase in immobility with longer time spent immobile per episode.

### Re-exposure Drinking

**FIGURE 6:**
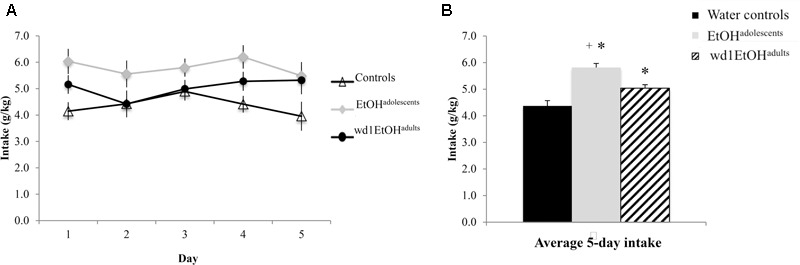
Increased consumption in alcohol-experienced animals. **(A)** Across the 5-day drinking period following behavioral testing, all alcohol-experienced animals consumed more alcohol than first-time drinkers. **(B)** When averaged across day, EtOH^adolescent^ consumed significantly more than wd1EtOH^adults^. ^∗^*p* < 0.05 vs. water controls, ^+^*p* < 0.05 vs. wd1EtOH^adults^. Data represent mean ± SEM, *n* = 9/group.

During the subsequent 5-day drinking period following behavioral testing, the repeated measures ANOVA showed a significant effect of prior alcohol experience [*F*(2,24) = 20.92, *p* < 0.001; **Figures 6A,B**]. *Post hoc* tests showed that both wd1EtOH^adults^ and EtOH^adolescents^ consumed more alcohol than first-time drinkers [wd1EtOH^adults^ *p* = 0.034, EtOH^adolescents^ *p* < 0.001]. Additionally, EtOH^adolescents^ drank more than wd1EtOH^adults^ (*p* = 0.003). Both wd1EtOH^adults^ and EtOH^adolescents^ also exhibited higher average alcohol consumption overall compared to their previous 14-day average [EtOH^adolescents^ *t*(8) = 3.53, *p* = 0.001, wd1EtOH^adults^ *t*(8) = 7.12, *p* < 0.001; Bonferroni α = 0.025]. There was no difference in intake between wd28EtOH^adults^ and PND98 water control animals [wd28EtOH^adults^: *M* = 3.63, SEM = 0.14; PND98 water controls: *M* = 3.09, SEM = 0.11; *t*(16) = 1.73, *p* = 0.10] and no increase in intake between the 14- and 5-day drinking period in wd28EtOH^adults^ [*t*(8) = 1.85, *p* > 0.10].

### Immunoblotting

**FIGURE 7:**
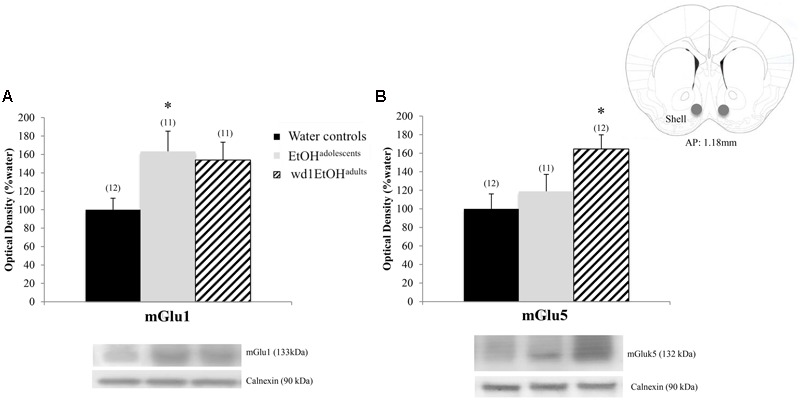
Alcohol-induced increases in mGluR expression within the AcbSh. **(A)** EtOH^adolescents^ showed a significant increase in mGlu1 expression within the AcbSh, with a similar trend in wd1EtOH^adults^. **(B)** wd1EtOH^adults^ showed a significant increase in mGlu5, with no change observed in EtOH^adolescents^. ^∗^*p* < 0.05 vs. water controls. Data represent mean + SEM of the number of animals indicated in parentheses.

In the AcbSh, there were significant group differences in mGlu1 expression [*F*(2,31) = 3.71, *p* = 0.03; **Figure 7A**] and mGlu5 [*F*(2,32) = 4.15, *p* = 0.02; **Figure 7B**]. *Post hoc* analysis showed that EtOH^adolescents^ had increased mGlu1 expression relative to water controls (*p* = 0.04), with a similar trend seen in wd1EtOH^adults^ (*p* = 0.09). wd1EtOH^adults^, but not EtOH^adolescents^, showed a significant increase in mGlu5 expression (*p* = 0.02 and *p* > 0.10, respectively). There were no group differences in Homer2b expression within the AcbSh (non-significant immunoblotting results are from the AcbSh and CeA are summarized in **Table 4**).

**Table 4:** Summary of non-significant immunoblotting results.

	PND70 water controls	EtOH^adolescents^	wd1EtOH^adults^	PND98 water controls	wd28EtOH^adults^
AcbSh: mGlu1				100 ± 16.33	97.33 ± 16.59
AcbSh: mGlu5				100 ± 21.07	117.34 ± 25.34
AcbSh: Homer2	100 ± 24.36	66.62 ± 8.07	126.86 ± 21.00	100 ± 16.94	73.78 ± 9.97
CeA: mGlu1				100 ± 32.63	76.48 ± 17.69
CeA: mGlu5	100 ± 12.12	198.81 ± 18.27	185.46 ± 14.76	100 ± 26.03	108.69 ± 22.60
CeA: Homer2				100 ± 24.78	71.31 ± 17.98
AcbC: mGlu1	100 ± 15.63	105.86 ± 15.90	92.05 ± 12.24	100 ± 18.14	80.39 ± 20.88
AcbC: mGlu5	100 ± 18.53	108.31 ± 24.17	113.36 ± 31.79	100 ± 11.30	115.52 ± 12.96
AcbC: Homer2	100 ± 19.42	116.98 ± 24.87	107.71 ± 18.91	100 ± 24.12	93.65 ± 13.10
BLA: mGlu1	100 ± 19.38	85.35 ± 17.36	116.25 ± 20.89	100 ± 15.88	81.98 ± 17.36
BLA: mGlu5	100 ± 14.07	75.80 ± 17.45	92.44 ± 17.37	100 ± 14.15	128.47 ± 26.05
BLA: Homer2	100 ± 13.28	86.09 ± 14.85	94.76 ± 16.15	100 ± 14.02	84.39 ± 15.06

*There were no significant differences in protein expression in adult drinkers following 28-days withdrawal (wd28EtOH^*adults*^) compared to age-matched water control animals. Data represent mean ± SEM, *n* = 10–11/group.*

**FIGURE 8:**
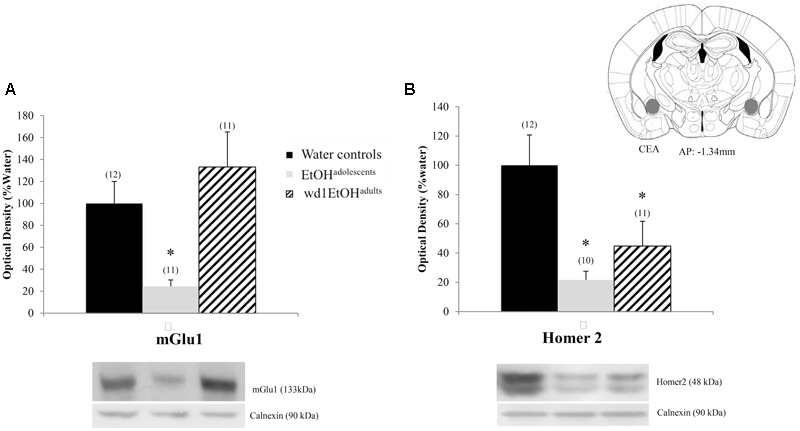
Alcohol-induced decreases in glutamate-related protein expression within the CeA. **(A)** EtOH^adolescents^, but not wd1EtOH^adults^, showed a significant decrease in mGlu1 expression within the CeA. **(B)** Both EtOH^adolescent^ and wd1EtOH^adults^ showed a significant decrease in homer 2 expression ^∗^*p* < 0.05 vs. water controls. Data represent mean + SEM of the number of animals indicated in parentheses.

There were significant group differences in mGlu1 expression within the CeA [*F*(2,33) = 6.32, *p* = 0.005; **Figure 8A**] and Homer 2b [*F*(2,30) = 5.97, *p* = 0.007; **Figure 8B**]. *Post hoc* testing showed that both EtOH^adolescents^ and wd1EtOH^adults^ had decreased Homer2b expression relative to water controls (*p* = 0.007 and *p* = 0.04, respectively). EtOH^adolescents^, but not wd1EtOH^adults^, showed a significant decrease in mGlu1 relative to water controls (*p* = 0.04 and *p* = 0.53, respectively). There were no group differences found in mGlu5 expression (**Table 4**). There were no significant differences in mGlu1, mGlu5, or Homer2 within the AcbC or BLA (**Table 4**; all *p*’s > 0.10).

**FIGURE 9:**
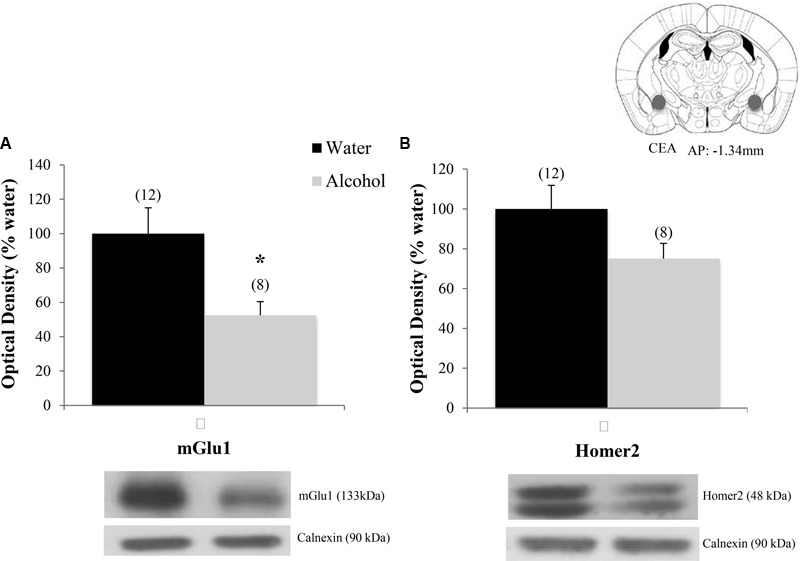
Decreases in glutamate-related protein expression within the CeA following single-bottle drinking. In a 14-day pilot study of single-bottle (20% EtOH) drinking in adults, animals consumed an average of 3.12 ± 0.18 g/kg. **(A)** Alcohol drinkers showed a significant decrease in mGlu1 expression within the CeA at 24 h withdrawal. **(B)** A similar negative trend was observed in Homer 2 expression. ^∗^*p* < 0.05 vs. water controls. Data represent mean + SEM of the number of animals indicated in parentheses. Unequal sample sizes due to sample availability, not outlier exclusion.

Finally, when the immunoblotting data for the adult mice drinking under our single-bottle paradigm were compared, we replicated the reduction in CeA expression of mGlu1 (**Figure 9A**) [*t*(18) = 3.05, *p* = 0.006] in alcohol-experienced animals versus water-drinking controls (**Figure 9A**) and also observed a trend toward reduced CeA Homer2 expression (**Figure 9B**) [*t*(18) = 1.86, *p* = 0.079].

## Discussion

### Drinking-Age-Dependent Behavioral Differences during Withdrawal

In prior work, we showed that adult mice with a binge-drinking history exhibit robust negative affect in the light-dark box, marble burying test, and FST during early (24 h) withdrawal that are not apparent in adolescent drinkers (Lee et al., 2016). In the present study, we assayed the behavior of adolescent drinkers during protracted withdrawal and uncovered distinct age-related differences in the time-course and presentation of withdrawal-induced negative affect in adolescent versus adult drinkers. Replicating our previous findings, wd1EtOH^adults^ showed signs of hyperanxiety during early withdrawal, as indicated by increased marble burying and decreased immobility in the FST. We have consistently observed decreased immobility in adult drinkers during early withdrawal, which we have interpreted as anxiety-related hyperactivity in response to an acute stressor (Lee et al., 2015, 2016, 2017). wd1EtOH^adults^ also showed increased sucrose preference compared to water control animals, which is not surprising given that studies have shown increased preference for sweet/sugary drinks amongst both humans (Kampov-Polevoy et al., 1997; Kranzler et al., 2001) and animals (Katz, 1982; Gosnell and Krahn, 1992; Stewart et al., 1994) with a history of chronic alcohol consumption. These results support the presence of hyperanxiety, but not depression, in wd1EtOH^adults^. However, these alcohol-induced behavioral differences dissipated during the course of withdrawal and by day 28, wd28EtOH^adults^ showed no significant differences compared to PND98 water control animals. This latter finding is particularly interesting as we reported previously that a 30-day history of binge-drinking during adulthood produces a persistent increase in negative affect across a large number of assays and behavioral measures (Lee et al., 2015). As the drinking period employed in this study was only 14 days, our collection of work indicates that not only the severity (Lee et al., 2016), but also the persistence, of alcohol withdrawal-induced hyper-anxiety varies as a function of the chronicity of binge alcohol-drinking in adults, with more chronic drinking experience eliciting more robust and enduring pharmacodynamic changes that drive the elevated negative affective state.

In contrast to adults with a 2-week binge-drinking history, EtOH^adolescents^ exhibited signs of both hyperanxiety and depression during protracted withdrawal. In fact, EtOH^adolescents^ demonstrated increased burying behavior across all measures in the marble-burying test and exhibited greater immobility in the FST, relative to wd1EtOH^adults^. Although general locomotion was not assessed in this study, it is unlikely that the FST results are attributable to suppressed locomotor activity, given the vigorous burying behavior exhibited in the marble-burying test. Based on conventional interpretations of the FST, this increased immobility is indicative of depressive-like behavior. Consistent with this interpretation, EtOH^adolescents^ also showed significantly lower sucrose preference relative to both wd1EtOH^adults^ and water control animals, supporting the presence of an anhedonic state. Interestingly, the difference in sucrose preference between EtOH^adolescents^ and wd1EtOH^adults^ suggests that an alcohol-induced preference for sweet liquids is either absent in EtOH^adolescents^ or is masked by the manifestation of anhedonia.

All alcohol-experienced animals consumed significantly more alcohol during the subsequent 5-day drinking period compared to their 14-day baseline average. Interestingly, EtOH^adolescents^ consumed significantly more than wd1EtOH^adults^, despite the fact that wd1EtOH^adults^ were earlier in withdrawal, when the presence of an alcohol deprivation effect is typically more pronounced (Melendez et al., 2006; Vengeliene et al., 2014). These data provide additional evidence that early alcohol experience predisposes individuals to higher alcohol consumption in adulthood and may thus accelerate the transition to chronic alcohol abuse and addiction.

The present data, combined with our prior work (Lee et al., 2016), argue that a history of binge-drinking during adolescence *does* elicit a robust negative affective state, but that the manifestation of this state is dependent upon an incubation period during withdrawal. These results are consistent with others reported in the preclinical literature. For example, Pandey et al. (2015) showed increased anxiety-like behavior in the light-dark box and elevated-plus maze and excessive alcohol consumption in rats at approximately 50 days withdrawal following adolescent alcohol exposure. In contrast to our previous findings, this prior study also showed evidence of increased anxiety at 24 h withdrawal in adolescent animals. However, given that alcohol was administered via IP injection, it is possible that there was an alcohol × stress interaction due to the stress related to the route of alcohol delivery.

Although the dissipation of withdrawal signs in wd28EtOH^adults^ during protracted withdrawal could be attributed to the relatively short 14-day drinking history, as our lab and others have shown persistent dysfunction following more prolonged alcohol exposure (Valdez et al., 2003; Santucci et al., 2008; Lee et al., 2015). However, this difference nonetheless demonstrates that, compared to adults, adolescent drinkers are hypersensitive to persistent dysfunction following even brief periods of binge-drinking. Such findings suggest that the neural dysfunction underpinning the emotional hyper-reactivity observed in adult mice with a prior adolescent drinking history undergoes an incubation- or sensitization-like process, which likely relates to alterations in the developmental trajectory of corticofugal afferents governing emotional control.

### Changes in Glutamate-Related Protein Expression within the AcbSh and CeA

Consistent with previous immunoblotting studies, wd1EtOH^adults^ showed increased mGlu5 expression in the AcbSh at 24 h withdrawal, with a similar positive trend in mGlu1 (Obara et al., 2009; Cozzoli et al., 2014; Lee et al., 2016). Although adolescent binge-drinkers do not exhibit increased Group 1 mGluR expression in early withdrawal (Lee et al., 2015), adolescent drinkers in the present study showed a significant increase in mGlu1, but not mGlu5, during protracted withdrawal. These results are consistent with evidence implicating the importance of Group 1 mGluRs within the AcbSh in drug-taking, including the positive reinforcing properties of alcohol (Gass and Olive, 2008), as well as the initiation, maintenance, and escalation of intake (Cozzoli et al., 2009, 2012, 2015; Kalivas et al., 2009; Griffin et al., 2014; Lum et al., 2014). Therefore, these changes could underlie the increased alcohol consumption seen during the subsequent 5-day drinking period. However, given that these protein changes coincided with the emergence of behavioral dysfunction, increased group 1 mGluR expression could also be relevant to withdrawal-induced negative affect. Additionally, the lack of differences in the AcbC and BLA demonstrate that these changes in protein expression are specific to extended amygdala subregions implicated in emotion.

The AcbSh receives significant glutamatergic input from the amygdala, which is known to mediate many of the negative reinforcing properties of alcohol withdrawal (Christian et al., 2012; Gilpin et al., 2015). Additionally, the Acb itself also has a role in negative affective states (Salamone, 1994; Shirayama and Chaki, 2006; Lim et al., 2012). There has been increased interest in the role of glutamatergic signaling within the Acb in aversive states such as anxiety, depression, and withdrawal-induced negative affect. For example, it has also been shown that an intra-AcbSh glutamate microinjection increases signs of depression in the FST, while inhibiting glutamate is antidepressant (Rada et al., 2003). Glutamatergic antagonism also alleviates the depressive, hypo-dopaminergic state during alcohol withdrawal (Rossetti et al., 1991). Therefore, the alcohol-induced increase in mGluR protein expression shown in the present study could render the AcbSh hypersensitive to glutamate-induced perturbation.

Within the CeA, EtOH^adolescents^ exhibited decreased mGlu1 expression and both EtOH^adolescents^ and wd1EtOH^adults^ showed decreased Homer2b expression during withdrawal. While these results are consistent with post-mortem studies in human alcoholics demonstrating reduced glutamate receptor isoform expression within the CeA (Jin et al., 2014), they contrast with published data from our group (Obara et al., 2009; Cozzoli et al., 2014) and others (e.g., Rossetti and Carboni, 1995; Roberto et al., 2004; Zhu et al., 2007) indicating an increase in glutamate-related signaling within the CeA during alcohol withdrawal. Comparable to our findings in the Acb, there were no significant changes in the BLA control region. This is consistent with previous studies from our lab (Obara et al., 2009; Cozzoli et al., 2014) and further substantiates the regional specificity of the changes observed herein. At the present time, it remains to be determined whether or not our inability to replicate our prior results from the CeA of binge-drinking C57BL/6J mice (i.e., Cozzoli et al., 2014) reflected procedural differences related to the total duration of alcohol-access (14 days vs. 30 days) or to the number of bottles presented during alcohol-access (4 vs. 1). However, the results of a pilot immunoblotting study in our laboratory suggest the former, as a 2-week history of access to a single 20% alcohol bottle also reduced mGlu1 within the CeA of wd1EtOH^adults^ at 24 h withdrawal, with a similar negative trend in Homer2 (**Figure 9**).

The functional relevance of the observed reduction in CeA mGlu1/Homer2 expression remains to be determined, particularly considering that negative affect is classically associated with amygdalar hyperactivation (Davis and Whalen, 2001; Shackman and Fox, 2016). However, optogenetic evidence supports a causal relationship between reduced glutamatergic signaling within the CeA and negative affective states (Tye et al., 2011). Under basal conditions, glutamatergic inputs from the BLA excite GABAergic medium spiny neurons within the lateral subdivision of the CeA, which in turn exerts feed-forward inhibition onto the adjacent medial subdivision of the CeA, the output region which mediates autonomic and behavioral responses associated with anxiety and fear through projections to the brainstem (Hilton and Zbrozyna, 1963; LeDoux et al., 1988; Davis and Whalen, 2001; Gilpin et al., 2015). Inhibition of this BLA projection reduces glutamatergic input to the CeA and increases anxiety-related behaviors, whereas stimulation of this projection is anxiolytic (Tye et al., 2011). Additionally, low glutamatergic input produces asynchronous firing of GABAergic neural networks within the amygdala (Zhang et al., 2012). This asynchronous firing is associated hyper-anxious behaviors that can be reversed by treatment with a group 1 mGluR agonist, which restores both neuronal synchronicity within the CeA and emotionality.

As the present study assayed protein expression in whole-cell homogenates, the site-specificity of these changes (i.e., subcellular location or cell phenotype) remains to be determined. Nevertheless, the work of Tye et al. (2011) and Zhang et al. (2012) support the possibility that reduced glutamate-related protein expression within the CeA, induced by a 2-week history of binge-drinking, may contribute to the manifestation of a hyper-anxious state in adult mice during early withdrawal. Furthermore, such a cause-effect relationship suggests that a time-dependent reduction in mGlu1/Homer2b-signaling within this region contributes to the apparent incubation of negative affect in mice with a prior history of binge-drinking during adolescence. In support of this possibility, no changes in glutamate receptor expression were observed within either the AcbSh or CeA in binge-experienced adult mice during protracted withdrawal (i.e., at a time when affective responding has normalized). As such, neuropharmacological and site-directed transgene delivery studies are currently on-going in our laboratory to directly assess the functional relationship between reduced glutamate signaling within the CeA and alcohol withdrawal-induced hyper-emotionality within the context of short-term binge-drinking.

## Conclusion

This study provides further basic science evidence to support a causal relationship between adolescent binge-drinking and negative outcomes manifested during protracted withdrawal in adulthood. Despite apparent insensitivity to the negative affective consequences of drinking during acute withdrawal, this study indicates that adolescent binge-drinkers are uniquely vulnerable to the latent maladaptive effects of alcohol upon emotionality that manifest in later withdrawal and shows that even a 2-week history of binge-drinking during the adolescent phase of neurodevelopment can have profound and enduring effects upon negative affect and subsequent drinking behavior, which are temporally related to molecular anomalies within brain regions regulating emotionality and negative reinforcement. This combination of negative affect and increased drinking likely contributes to the predisposition toward alcohol abuse and alcoholism later in life. Alcohol-induced dysregulation within extended amygdala structures regions offers a potential neurobiological correlate for the high comorbidity between substance abuse and mood disturbances. Additional research is necessary to characterize the progression and duration of these changes throughout the course of withdrawal in order to further our understanding of the ontogenetic differences in the etiology of alcoholism and its high rate of comorbidity with affective disorders.

## Ethics Statement

All of the research described in this report was approved by the Institutional Animal Care and Use Committee of the University of California, Santa Barbara.

## Author Contributions

KL, MC, NS, and KS conducted the experiments. KL and KS analyzed the data. KL composed the manuscript. MC, NS, and KS edited the manuscript.

## Conflict of Interest Statement

The authors declare that the research was conducted in the absence of any commercial or financial relationships that could be construed as a potential conflict of interest.
